# Supplementary material for: Perceptions and Feasibility of Actions Related to Sodium Reduction among Restaurant Owners and Cooks in Seongnam, South Korea: Comparison According to Stages of Behavioral Change
Source: Nutrients. 2021 Dec 6;13(12):4375. doi: 10.3390/nu13124375 (PMC8707999; doi:10.3390/nu13124375)
Supplement: Supplementary file 1 [file nutrients-13-04375-s001.zip › nutrients-1476374-supplementary.pdf]

Thank you for participating this survey. It aims to collect basic data for ‘Making a healthy table project’.

Your response will be only used for survey purposes and all your answers will be held in the strictest of confidentially.

Please put a tick in the box ☑ next to the answer of your choice or write in the space provided.

1. Please fill out the following for your personal information and workplace information.

Gender	☐ Male	☐ Female
Age	( ) years old	
Height	( ) cm	
Weight	( ) kg	
Location of restaurant	☐ Bundang-gu<br>☐ Sujeong-gu<br>☐ Jungwon-gu	
Position in restaurant	☐ Owner<br>☐ Cook<br>☐ Owner and cook	
Types of food served	☐ Home-style Korean meal<br>☐ Grilled meat (Pork belly, rib..)<br>☐ Japanese food<br>☐ Chinese food<br>☐ Others	☐ Western food<br>☐ Snack food<br>☐ Burger, pizza, fried chicken<br>☐ Bakery (sandwich, toast...)
Size of restaurant	☐ Under 100 m^2<br>☐ 100 m^2 - 300 m^2<br>☐ Above 300 m^2	
Operating system of restaurant	☐ Independently owned<br>☐ Chain restaurant	

2. Do you recognize nutrition education program, campaign or events for reducing sodium intake on TV, radio, advertisement, and in the newspaper?

   ☐ Yes ☐ No

3. Have you seen or heard about sodium labeling on foods in restaurant or highway rest area?

   ☐ Yes ☐ No

4. Are you currently practicing sodium reduction in your restaurant?

   ☐ Yes, more than 6 months

   ☐ Yes, but less than 6 months

   ☐ No (Please, answer the next question)

1-1. Do you intend to make changes to reduce sodium use in your restaurant in the near future?

☐ Yes, in the following month,

☐ No, but in the next 6 months,

☐ No, I haven't thought about it.

5. Is there salt or salty sauce on the table in your restaurant?
   ☐ Yes ☐ No

6. Is the amount of salt and salty condiments measured while cooking in your restaurant?
   ☐ Almost always ☐ Often ☐ Scarcely

7. Is the salinity of food measured in your restaurant?
   ☐ Almost always ☐ Often ☐ Scarcely

8. Is the salinity information of food provided in your restaurant?
   ☐ Yes ☐ No

9. What advantages do you expect by reducing sodium use in restaurant? (select main factors)
   ☐ Improve the restaurant's image
   ☐ Help to take care of health of customers and employees
   ☐ Make the food taste better by revealing the natural flavor in food
   ☐ Satisfy customers' varied preference for saltiness
   ☐ Help to develop menu by having more interest in cooking method
   ☐ Other opinion ( )

10. What difficulties do you expect when you practice sodium reduction in restaurant? (select main factors)
   ☐ Hard to maintain taste
   ☐ Time-consuming and inconvenient process of cooking
   ☐ Limited knowledge and skills to practice
   ☐ Limitation to choose food items for low-sodium dishes
   ☐ High cost
   ☐ Complaint of customers
   ☐ Short shelf-life and spoilage of foods
   ☐ Other opinion ( )

11. Are you willing to reduce sodium use in restaurant under the support of government?

   ☐ Yes ☐ No

12. What kind of support do you think is needed if government provide support to reduce sodium in restaurant?
   ☐ Publicizing participating restaurants
   ☐ Providing plaques of 'healthy restaurant' to participating restaurant
   ☐ Providing salimeter and educating how to use
   ☐ Analyzing sodium content and support to display it
   ☐ Training cooking skills to reduce salt use
   ☐ Support for educating employees

13. Please rate the following statements of actions in restaurant.

	Very difficult	Difficult	Neither difficult nor easy	Easy	Very easy
Purchase foods after comparing sodium content in nutrition labels	☐	☐	☐	☐	☐
Measure the amount of salt and salty condiments while cooking	☐	☐	☐	☐	☐
Use salimeter and keep the standard salinity	☐	☐	☐	☐	☐
Cook with low-sodium sauce or broth	☐	☐	☐	☐	☐
Use herbs and spices to reduce the amount of MSG	☐	☐	☐	☐	☐
Apply cooking skills to make low- sodium dishes	☐	☐	☐	☐	☐
Make food less salty and serve sauce separately	☐	☐	☐	☐	☐
Offer some menus with less salty options that customers can choose	☐	☐	☐	☐	☐
Add low-salt menu	☐	☐	☐	☐	☐
Offer optional side menu with fresh vegetables and fruits	☐	☐	☐	☐	☐
Avoid serving salt-fermented foods as side dishes	☐	☐	☐	☐	☐
Serve less salty kimchi	☐	☐	☐	☐	☐
Serve small portions of kimchi	☐	☐	☐	☐	☐
Display sodium content of foods served	☐	☐	☐	☐	☐
Put up promotional materials for reducing sodium intake	☐	☐	☐	☐	☐

*Thank you for completing the survey.
